# Response-repetition costs in task switching do not index a simple response-switch bias: Evidence from manipulating the number of response alternatives

**DOI:** 10.3758/s13414-023-02708-2

**Published:** 2023-05-05

**Authors:** Iring Koch, Eliot Hazeltine, Greta Petersen, Daniel H. Weissman

**Affiliations:** 1 Institute of Psychology, RWTH Aachen University, Jägerstr. 17-19, D-52056 Aachen, Germany; 2 Department of Psychological and Brain Sciences, University of Iowa, Iowa City, IA USA; 3 Department of Psychology, University of Michigan, Ann Arbor, MI USA

**Keywords:** Cognitive and attentional control, Repetition effects, Task switching or executive control

## Abstract

Response repetitions aid performance when a task repeats but impair performance when a task switches. Although this interaction is robust, theoretical accounts remain controversial. Here, we used an un-cued, predictable task-switching paradigm with univalent targets to explore whether a simple bias to switch the response when the task switches can explain the interaction. In Experiment 1A (n = 40), we replicated the basic interaction in a two-choice task. In Experiment 1B (n = 60), we observed the same interaction in a three-choice task, wherein a bias to switch the response when the task switches cannot prime a specific alternative response because both remaining response alternatives are equally likely. Exploratory comparisons revealed a larger interaction between task repetition and response repetition in the three-choice task than in the two-choice task for mean response time (RT) and the opposite pattern for mean error rate (ER). Critically, in the three-choice task, response-repetition costs in task switches were significant in both RT and ER. Since a bias to switch the response cannot prime a specific response alternative in a three-choice task, we conclude that such a bias cannot account for response-repetition costs in task-switch trials.

## Introduction

Cognitive control enables flexible action control in rapidly changing contexts. Researchers often investigate such control by asking participants to switch between two or more tasks. Performance is consistently worse in task switches than in task repetitions, which has been termed a “switch cost” (for reviews, see Kiesel et al., 2010; Koch et al., 2018b; Koch & Kiesel, 2022; Monsell, 2003).

Another robust finding is that task switching modulates the effects of repeating a previous response on performance. For example, Rogers and Monsell (1995) introduced a two-choice paradigm in which a letter and a digit appeared simultaneously on the computer screen. Here, participants alternated predictably between digit classification (odd or even) and letter classification (consonant or vowel) in an AABB task sequence using two response keys that are shared between the two tasks. The authors found that repeating the response from one trial to the next aided performance if the task repeated, but impaired performance if the task switched. This crossover interaction is very robust (see Gade et al., 2014, for a review). It appears in predictable tasks (Kleinsorge, 1999), explicitly cued tasks (Meiran, 2000), with auditory stimuli (Quinlan, 1999; Seibold et al., 2019), with manual responses within a hand versus across hands (Koch et al., 2011), and even across different effectors (i.e., manual vs. vocal; Schuch & Koch, 2004). Yet, there is no consensus about the underlying mechanisms. As we describe next, there are three main accounts for this crossover interaction.

## Three accounts of response-repetition effects in task switching

The *inhibition account* assumes a bias to prevent unwanted response perseveration when tasks change quickly. Here, an inhibitory mechanism targets the just-executed response when the task switches to prevent a response repetition. Response inhibition also occurs when the task repeats. Yet, because response repetitions are associated with repetitions of the stimulus category (or the stimulus itself), inhibition is overshadowed by strong, positive category priming. Thus, response repetitions produce a net benefit to performance when the task repeats (Druey, 2014; Druey & Hübner, 2008; Hübner & Druey, 2006; see Gade et al., 2014).

The *associative interference* account assumes an association between the current task and the current response that aids performance when both repeat but impairs performance when one repeats and the other changes. Specifically, while repeating both the task and the response facilitates the correct response, repeating one (e.g., the task) while switching the other (e.g., the response) creates associative interference (Meiran, 2000; Rogers & Monsell, 1995). One variant of this account assumes that responses are coded in terms of their task-specific “meaning” (e.g., “left key means consonant”), so that the meaning of the motor response needs to be recoded in a task switch, which produces recoding costs (see Schacherer & Hazeltine, 2022; Schuch & Koch, 2004).

Another version of this account refers to the influence of *feature binding* on performance. Here, the task and the response are treated as independent features bound together in an event file (Schuch & Koch, 2004). Repeating the task triggers the retrieval of the previous response, which aids performance when the response repeats but impairs performance when the response switches. The latter “partial repetition cost” (Hommel, 1998; Hommel et al., 2001) occurs because repeating the previous task (e.g., classify digits) triggers the retrieval of the previous response (e.g., left keypress), which conflicts with the current response (e.g., right keypress). This conflict takes time to resolve, as it is necessary to overcome the previous task-response association to associate the previous task with a new response (see Altmann, 2011; Kandalowski et al., 2020; Koch et al., 2018a; Schuch & Koch, 2004, 2006, 2010; see Frings et al., 2020, for a recent review of feature binding in action control). In contrast, selecting a response in trials with complete feature mismatch (i.e., a response switch in a task-switch trial) is not hampered by additional retrieval conflict.

Finally, the *bias to switch responses* account posits that participants acquire a heuristic bias to switch the response whenever the situation changes (e.g., Fletcher & Rabbitt, 1978), resulting in a response-repetition cost if the stimulus repeats (see also Williams, 1966, Experiment 4). Kleinsorge (1999) demonstrated such a cost in a variant of the alternating runs procedure developed by Rogers and Monsell (1995). In his Experiments 2 and 3, participants responded to letters or symbols with two response keys. Repeating the stimulus set resulted in a substantial response-repetition benefit, while switching the stimulus set abolished this response-repetition benefit in reaction time (RT) and reversed it in error rates.

Kleinsorge and Heuer (1999) observed similar effects in a more complex study. These authors employed a numerical and a spatial judgment task but combined this with a stimulus-response mapping manipulation (compatible vs. incompatible) that changed independently. In this study, repeating the mapping was beneficial only when the judgment task repeated. Furthermore, repeating the response was beneficial only when both the response mapping and the judgment task repeated. To explain this complex data pattern, the authors proposed a hierarchical control account, in which a switch at a higher level of the control structure automatically propagates downstream to lower levels. According to this account, any change to the task (i.e., classification, response mapping, etc.) at a higher hierarchical level engenders a bias to switch the response at lower hierarchical levels. This account implements the bias to switch responses account of response-repetition costs into a model of task control that is applicable to situations requiring frequent and flexible task switching.

Although these three accounts differ strongly in terms of the underlying theoretical assumptions, they make similar predictions. Consequently, it has proven difficult to adjudicate among them. Moreover, the proposed mechanisms are not necessarily mutually exclusive (e.g., Koch et al., 2018a). Hence, explaining the pattern of response-repetition costs and benefits in task switching remains a theoretical challenge.

## A potentially informative variable: The number of response alternatives

Almost all task-switching studies on response-repetition effects have used two tasks and two response options (with few exceptions; see, e.g., Kikumoto & Mayr, 2020). Under these conditions with binary response choice, it is easy to see how a strategic bias to switch the response when the task or stimulus switches could facilitate a response switch – leading to response-repetition costs – because there is only one response alternative. In this scenario, the three accounts above make clear predictions, and they can all explain the crossover interaction between task repetition and response repetition. Therefore, in the present study, we examined response-repetition effects in task switching with more than two response alternatives. We did this to compare the pattern of response-repetition effects with two response alternatives to that with three response alternatives. Although all three accounts predict that the response-repetition benefit in task repeat trials will be unaffected by the number of response alternatives, one of the three accounts appears to make a different prediction to the other two with respect to response-repetition costs in task-switch trials.

What does each account predict with respect to response-repetition costs in task-switch trials in a three-choice task? The inhibition account predicts that switching tasks will continue to engender inhibition of the just-executed response leading to significant response-repetition costs. The associative account described in terms of feature binding predicts that repeating a response while switching tasks will continue to engender partial repetition costs due to associative interference, creating retrieval conflict and leading to significant response-repetition costs. In contrast, the bias to switch responses account predicts that switching tasks will no longer engender response-repetition costs. The reason is that switching tasks does not cue a specific response alternative in a three-choice task wherein there are two possible response alternatives, rather than just one.

## The present study

In light of the considerations above, we investigated whether response-repetition costs in task-switch trials appear in a two-choice task but vanish in a three-choice task. We reasoned that observing such an outcome would be consistent with the bias to switch responses account but less consistent with the two other accounts. In contrast, not observing this outcome would be consistent with the other two accounts but harder to reconcile with the bias to switch account.

To isolate the influence of the number of response alternatives on response-repetition effects in task switching, we reduced the complexity of typical task-switching paradigms in two ways. First, rather than using bivalent stimuli – such as the simultaneous presentation of a letter and a digit in Rogers and Monsell’s (1995) study – we used univalent stimuli that are associated with only a single task. Second, as in Rogers and Monsell (1995), we used a predictable, alternating-runs task sequence that does not require presenting an explicit task cue prior to each target.

We chose not to use bivalent stimuli because they create congruency effects. Because bivalent stimuli are associated with both tasks, processing them can lead to the activation of one response (congruent stimuli) or two responses (incongruent stimuli). This introduces unnecessary complexity for two reasons. First, response-repetition costs are especially large when the previous trial is congruent (e.g., Grzyb & Hübner, 2013). Grzyb and Hübner (2013) suggested that congruent trials produce particularly strong response activation, which increases the risk of response perseveration and thereby increases inhibition targeted against the just-executed response. Second, congruency effects on the previous trial affect the size of congruency effects in the current trial (i.e., the congruency sequence effect; see Egner, 2007, for a review; see also Weissman et al., 2016), which might complicate the interpretation of response-repetition effects. For these reasons, we chose to employ univalent stimuli, thereby completely avoiding the issue of congruency. Although switch costs are smaller for univalent stimuli than for bivalent stimuli, response-repetition benefits have still been reported for task-repeat trials while response-repetition costs in task-switch trials remained significant (at least in error rates; see Rogers & Monsell, 1995; Experiment 4). Consequently, we expected a robust interaction between task repetition and response repetition in a two-choice task even for univalent stimuli (see also Kleinsorge, 1999).

Given that we decided to use univalent stimuli, it was not necessary to use explicit task cues. Independent of this consideration, however, we did not deem it desirable to use such cues. Our reasoning was that the cue itself can be viewed as a perceptual feature of the experimental trial that can repeat or switch, which may produce priming effects independent of repeating or switching the task (Altmann, 2011; Schneider & Logan, 2005). For example, Schneider and Logan (2005) reported that when using two cues for each task, the response repetition benefit in RT vanished when the alternate cue signaled the same task (i.e., a task repetition). While this might be consistent with an account wherein any stimulus change engenders a bias to switch the response, we deemed it useful to avoid the complexity of introducing perceptual cue repetitions and perceptual cue switches by simply omitting cues. Yet, to induce strong task representations, we still presented the tasks in a AABB sequence (alternating runs), so that task switches and repetitions were fully predictable.

Finally, to avoid carry-over effects, we examined the influence of the number of response alternatives on response-repetition effects in a between-subjects design. We report the data from our online two-choice and three-choice tasks as Experiment 1A and Experiment 1B, respectively, but we designed these tasks to allow direct comparisons.

## Experiment 1A (two-choice task)

Experiment 1A (two-choice task) was meant to demonstrate the basic interaction between task repetition and response repetition in an online setting. We used a set of four digits ([2, 3], [8, 9]) for a digit classification task (small vs. large numerical magnitude) and a set of four letters ([E, U], [K, Q]) for a letter classification task (vowel vs. consonant).

### Method

#### Participants

The experiment was conducted online during the period from December 2020 to January 2021 using Gorilla for hosting the experiment (Anwyl-Irvine et al., 2020). We recruited participants from the participant pool of the psychology program at RWTH Aachen University and via word of mouth. Participants had to be within an age range of 18–35 years. However, we did not collect demographic or gender information. Psychology students received partial course credit for participation. Data were collected from 42 participants, but data from two participants were excluded due to excessively long response times, resulting in a final sample of *N* = 40. A post hoc power analysis using GPower 3.1 (Faul et al., 2007) with alpha = 0.05 (two-tailed) yielded a power of .87 to detect a medium effect size (d_z_ = 0.5).

#### Stimuli and task

The experiment was programmed with PsychoPy2 and hosted using Gorilla experiment builder (Anwyl-Irvine et al., 2020). We used the digits 2, 3, 8, and 9 and the letters E, U, K, and Q. We created visual stimuli that we thought would be easy to perceive, but we could not standardize the presentation size due to the unknown sizes and resolutions of the participants’ computer screens in this online experiment. We assumed that small variations in stimulus size would not have a systematic effect on performance.

We instructed participants to press the B and M keys on their computer keyboard with the ring and index fingers of their right hand (leaving out the middle finger, which was relevant in Experiment 1B, see below). The digit classification task was a small (2 & 3) versus large (8 & 9) numerical magnitude classification mapped to the B and M keys, respectively. The letter classification task was a vowel (E & U) versus consonant (K & Q) letter classification mapped to the B and M keys, respectively.

#### Procedure

We presented the tasks such that each pair of digit trials followed a pair of letter trials (and vice versa) in an AABB task sequence. We divided the stimuli for each task into two subsets that appeared in alternating trials. For example, in consecutive digit task trials, we presented a stimulus from the digit subset 2 & 8 followed by a stimulus from the digit subset 3 & 9 (we drew stimuli randomly from within each set). We did this to ensure that (1) no direct stimulus repetitions occurred in consecutive trials and (2) response switches and response repetitions occurred equally often. We created four different versions of the subsets above, so that every combination could occur across participants in a counterbalanced way (Table 1 shows the list of possible subsets and the resulting possible combinations).

**Table 1:** Four possible combinations of stimulus subsets (Experiment 1A)

Alternating digit sets	Alternating letter sets
{2/ 8} & {3/ 9} {2/ 8} & {3/ 9} {2/ 9} & {3/ 8} {2/ 9} & {3/ 8}	{E/ K} & {U/Q} {E/ Q} & {U/ K} {E/ K} & {U/ Q} {E/ Q} & {U/ K}

Across participants, we counterbalanced whether a block of trials started with the letter task or the digit task. We also equated the number of response switches and response repetitions within blocks. In this procedure, it was possible for task-switch trials that a stimulus could re-occur as a lag3 repetition. As described below, we removed such trials, theoretically 25% of the task-switch trials, from data analyses to avoid such lag3 repetitions (which would represent direct target repetition with this task and if one disregarded the two intervening trials with the other task). This procedure also introduces a slight stimulus frequency imbalance between task-repeat and task-switch trials. For task-repeat trials, there are only two stimulus options because we draw stimuli from only a single subset. For task-switch trials, however, there are again four stimulus options because we draw stimuli from either of two subsets. This imbalance may favour task repetitions slightly (due to a lower number of stimulus alternatives). However, this is likely not critical for the consideration of response-repetition effects, because we equated the frequencies of response repetitions and response switches in task-switch and task-repeat trials (see Altmann, 2011, for a discussion).

The experiment started with a short practice block of 16 trials, in which each possible stimulus appeared twice. Subsequently, eight blocks of 64 trials appeared. The response-stimulus interval (RSI) was 500 ms. If a response was incorrect, error feedback (a red cross) was presented for 200 ms, extending the RSI to 700 ms. If a response was correct, no feedback was given. The experiment took approximately 15 min.

#### Design

The independent variables were task transition (switch vs. repeat) and response transition (switch vs. repeat). We analysed the predicted response-repetition effects in task switches and repetitions using one-tailed paired t-tests. The dependent variables were RT and error rates.

## Results

**Fig. 1:**
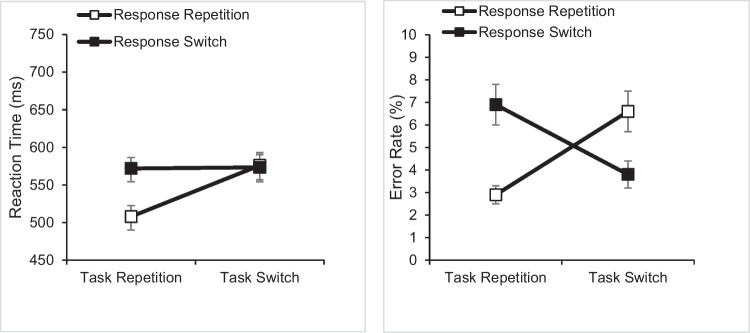
Mean reaction time (in ms; left panel) and error rate (in %; right Panel) in Experiment 1A (two response alternatives). Error bars represent standard errors

For the analyses of RTs and error rates, we removed the practice block, the first trial in each block, and trials after errors. In task-switch trials, we removed possible stimulus repetitions relative to the last trial with the same task (i.e., trial n-3). For RT analyses, we also excluded error trials. We filtered the remaining trials for outliers, defined as (a) RTs below 100 ms, (b) RTs > two standard deviations (SD) above the condition mean RT, and (c) RTs < two SD below the condition mean (altogether 4.1%). Figure 1 shows the RT (left panel) and error rate (right panel) as a function of task transition and response transition.

For RTs, we ran a 2 × 2 repeated-measures analysis of variance (ANOVA) with the independent variables task transition (task switch vs. task repetition) and response transition (response switch vs. response repetition). We observed a significant main effect of task transition (*F*(1, 39) = 90.016, *p* < .001, η_p_^2^ = .698) and of response transition (*F*(1, 39) = 32.738, *p* < .001, η_p_^2^ = .456). On average, mean RT was 35 ms shorter for task repetitions than for task switches (540 ms vs. 575 ms) and 31 ms shorter for response repetitions than for response switches (542 ms vs. 573 ms). Importantly, the expected interaction was significant (*F*(1, 39) = 118.130, *p* < .001, η_p_^2^ = .752). There was a 64-ms response-repetition benefit in task-repeat trials (*t*(39) = 8.777, *p* < .001, d_z_ = 1.388), but a non-significant, 3-ms response-repetition cost in task-switch trials (*t*(39) = -0.575, *p* < .568, d_z_ = 0.091).

For error rates, the effects of task transition (*F*(1, 39) = 0.730, *p* = .398, η_p_^2^ = .018) and response transition were not significant (*F*(1, 39) = 2.191, *p* = .147, η_p_^2^ = .053). However, the predicted interaction was significant (*F*(1, 39) = 44.358, *p* < .001, η_p_^2^ = .532). There was a 3.9% response-repetition benefit in task-repeat trials (*t*(39) = 5.746, *p* < .001, d_z_ = 0.909) and a 2.7% response-repetition cost in task-switch trials (*t*(39) = -4.566, *p* < .001, d_z_ = 0.722).

## Discussion

We obtained the expected two-way interaction pattern in RTs and error rates (Kleinsorge, 1999; Rogers & Monsell, 1995). Specifically, we observed a robust response-repetition benefit in task-repeat trials and a response-repetition cost in task-switch trials (even though it was significant only in error rate). This set the stage for determining whether the number of response alternatives alters response-repetition costs in task-switch trials. Hence, in Experiment 1B, we increased the number of response alternatives from two to three.

## Experiment 1B (three-choice task)

As we described in the *Introduction*, the three main accounts of the task repetition by response repetition interaction make similar predictions for task-repeat trials in a three-choice task but make different predictions for task-switch trials. For task-repeat trials, these accounts predict that the response-repetition benefit will be largely unaffected by number of response alternatives. For task-switch trials, however, the bias to switch responses account predicts diminished or even eliminated response-repetition costs while the other accounts continue to predict robust response-repetition costs.

### Method

#### Participants

We conducted Experiment 1B in April of 2021 as an online experiment hosted via Gorilla (just like Experiment 1A). The participants were recruited via Prolific (https://www.prolific.co/). The prerequisite to participate in the experiment was to be within an age range of 18–35 years. However, we did not collect demographic or gender information. Sixty-four participants were tested, but data from four participants were excluded from the analysis due to very high error rates (> 15%). This resulted in a sample of 60 participants. A (post hoc) power analysis using GPower 3.1 (Faul et al., 2007) with alpha = 0.05 yielded a power of 0.97 with an expected effect size of d_z_ = 0.5 and N = 60.

#### Stimuli, tasks, procedure, and design

Experiment 1B was similar to Experiment 1A except that for each task, we included two additional stimuli to create the third stimulus category and thus a third response option. For the digit task, we added two digits of medium magnitude (5 or 6) that required a response with the right middle finger. For the letter task, we added two non-alphabetical symbols (? and %) that required a response with the right middle finger. The two tasks still alternated in runs of two (i.e., in an AABB sequence), as in Experiment 1A, and we again formed different stimulus subsets for subsequent trials of the same task. However, we created alternating subsets with three stimuli (e.g., [2 & 5 & 8] and [3 & 6 & 9] or [E & K & ?] and [U & Q & %]), rather than just two, thus also reducing the probability of a response repetition from *p* = 0.5 to *p* = 0.33. This procedure resulted in 16 subsets for each task that included all possible stimulus-response combinations (Table 2). The 16 subsets for each task, together with whether a block started with the letter task or the digit task, resulted in 32 different counterbalancing versions.

**Table 2:** Sixteen possible combinations of stimulus subsets (Experiment 1B)

Alternating digit sets	Alternating symbol sets
{2/ 5/ 8} & {3/ 6/ 9} {2/ 5/ 8} & {3/ 6/ 9} {2/ 5/ 8} & {3/ 6/ 9} {2/ 5/ 8} & {3/ 6/ 9} {2/ 6/ 8} & {3/ 5/ 9} {2/ 6/ 8} & {3/ 5/ 9} {2/ 6/ 8} & {3/ 5/ 9} {2/ 6/ 8} & {3/ 5/ 9} {2/ 5/ 9} & {3/ 6/ 8} {2/ 5/ 9} & {3/ 6/ 8} {2/ 5/ 9} & {3/ 6/ 8} {2/ 5/ 9} & {3/ 6/ 8} {2/ 6/ 9} & {3/ 5/ 8} {2/ 6/ 9} & {3/ 5/ 8} {2/ 6/ 9} & {3/ 5/ 8} {2/ 6/ 9} & {3/ 5/ 8}	{E/ K/ ?} & {U/ Q/ %} {E/ Q/ ?} & {U/ K/ %} {E/ K/ %} & {U/ Q/ ?} {E/ Q/ %} & {U/ K/ ?} {E/ K/ ?} & {U/ Q/ %} {E/ Q/ ?} & {U/ K/ %} {E/ K/ %} & {U/ Q/ ?} {E/ Q/ %} & {U/ K/ ?} {E/ K/ ?} & {U/ Q/ %} {E/ Q/ ?} & {U/ K/ %} {E/ K/ %} & {U/ Q/ ?} {E/ Q/ %} & {U/ K/ ?} {E/ K/ ?} & {U/ Q/ %} {E/ Q/ ?} & {U/ K/ %} {E/ K/ %} & {U/ Q/ ?} {E/ Q/ %} & {U/ K/ ?}

The experiment started with a short practice block of 24 trials, in which each possible stimulus was presented twice. Subsequently, eight blocks of 72 trials (compared to 64 trials in Experiment 1A) were completed. The experiment took approximately 20 min.

## Results

**Fig. 2:**
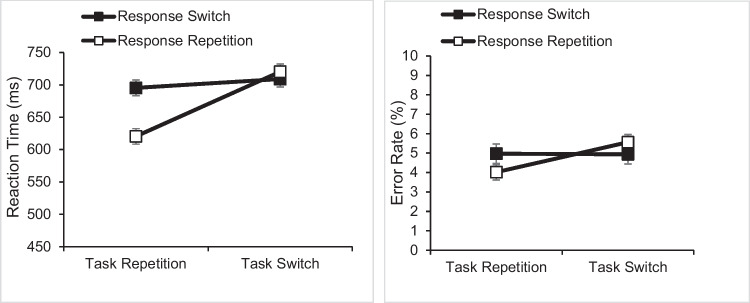
Mean reaction time (in ms; left panel) and error rate (in %; right panel) in Experiment 1B. Error bars indicate standard errors

We prepared the data for analysis as in Experiment 1A (e.g., we defined outliers as in Experiment 1A: altogether, 3.4%). Figure 2 shows mean RT and mean error rate as a function of task transition and response transition.

For RTs, the ANOVA showed a significant main effect of task transition (*F*(1, 59) = 171.019, *p* < .001, η_p_^2^ = .743) and of response transition (*F*(1, 59) = 40.105, *p* < .001, η_p_^2^ = .405). On average, mean RT was 59 ms shorter on task-repeat trials than on task-switch trials (658 ms vs. 717 ms), and mean RT was 30 ms shorter on response-repeat trials than on response-switch trials (672 ms vs. 702 ms). Critically, the interaction was significant (*F*(1, 59) = 126.381, *p* < .001, η_p_^2^ = .682). There was a significant 74-ms response-repetition benefit in task-repeat trials (*t*(59) = 9.987, *p* < .001, d_z_ = 1.289) and a significant 13-ms response-repetition cost in task-switch trials (*t*(59) = -2.862, *p* = .006, d_z_ = 0.369).

For error rates, there was a main effect of task transition (*F*(1, 59) = 8.796, *p* = .004, η_p_^2^ = .130), showing fewer errors in task-repeat trials than in task-switch trials (4.5% vs. 5.3%), but no main effect of response transition (*F*(1, 59) = 0.275, *p* = .602, η_p_^2^ = .005). Critically, the interaction between task transition and response transition was significant (*F*(1, 59) = 8.420, *p* = .005, η_p_^2^ = .125). There was a 0.95% response-repetition benefit in task-repeat trials (*t*(59) = 2.163, *p* = .035, d_z_ = 0.279), and a 0.6% response-repetition cost in task-switch trials (*t*(59) = -1.726, *p* < .045 (one-tailed), d_z_ = 0.223).

## Discussion

As in Experiment 1A, we found the interaction between task transition and response transition. Critically, response-repetition costs remained significant in task-switch trials in the error rates and were actually significant in the RTs (unlike in Experiment 1A). As explained earlier, this outcome favors the inhibition and associative interference accounts of the task transition by response transition interaction over the bias to switch responses account.

Broadly, the data from Experiment 1B are very similar to those from Experiment 1A. That is, we observed a pronounced cost-benefit pattern primarily in mean error rates, whereas the RT data showed a strong response-repetition benefit in task-repeat trials but only a small response-repetition cost in task-switch trials. While the pattern of RT data looks very similar across experiments, the interaction pattern in the error rates seems to be somewhat flattened in Experiment 1B relative to Experiment 1A. Therefore, to compare the data sets more formally, we ran additional between-experiment ANOVAs.

## Across-experiment comparisons

**Fig. 3:**
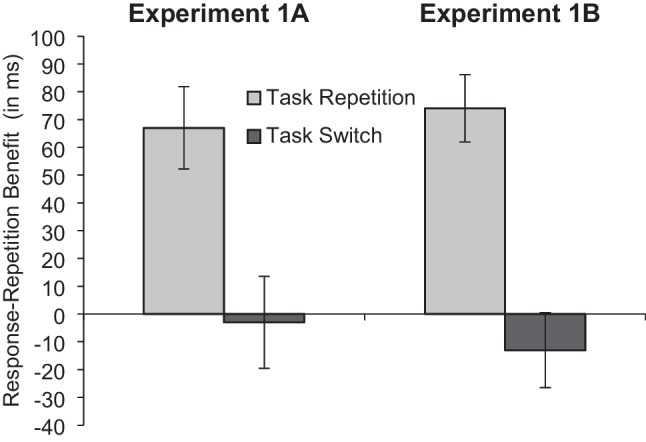
Response-repetition effects in reaction time (in ms) as a function of task transition and number of response alternatives (Experiment 1A vs. Experiment 1B). Mean values are shown; error bars are standard errors

The interaction in RT was slightly less pronounced in the two-choice task in Experiment 1A than in the three-choice task in Experiment 1B (see Fig. 3). In the two-choice task, there was a 64-ms response-repetition benefit in task-repeat trials and a 3-ms response-repetition cost in task-switch trials (i.e., an interaction contrast of 67 ms). In the three-choice task, however, there was a 74-ms response-repetition benefit in task-repeat trials and a 13-ms response-repetition cost in task-switch trials (an interaction contrast of 87 ms).

An across-experiment ANOVA showed the expected main effect of experiment, indicating that overall RT was 130 ms longer in Experiment 1B than in Experiment 1A (687 ms vs. 557 ms; *F*(1, 98) = 41.867, *p* < .001, η_p_^2^ = .299). This shows the general influence of the number of response alternatives (3 vs. 2) on response selection (see the *General discussion*). Here, we focus on effects that interacted with the experiment factor to avoid redundancy with the analyses presented earlier.

In particular, experiment interacted with task transition, indicating larger task-switch costs in the three-choice task than in the two-choice task (59 ms vs. 35 ms; *F*(1, 98) = 14.689, *p* < .001, η_p_^2^ = .130). The interaction of experiment with response transition was not significant (*F*(1, 98) = 0.001, *p* = .970, η_p_^2^ = .000). Yet, even though the three-way interaction was not significant, there was a trend toward a more pronounced task transition × response transition interaction effect in the three-choice task than in the two-choice task (87 ms vs. 67 ms; *F*(1, 98) = 3.566, *p* = .062, η_p_^2^ = .035).

**Fig. 4:**
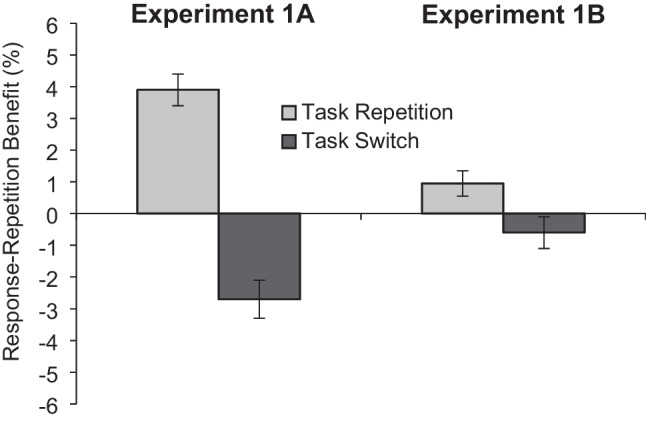
Response-repetition effects in error rate (in %) as a function of task transition and number of response alternatives (Experiment 1A vs. Experiment 1B)

Before attempting to speculate about the potential implications of this trend, it is important to note that the error rates showed the opposite pattern (Fig. 4). That is, in the error rates the two-way interaction was numerically larger in the two-choice task (3.9% response-repetition benefit in task-repeat trials plus 2.7% response-repetition cost in task-switch trials) than in the three-choice task (0.95% response-repetition benefits in task-repeat trials plus 0.6% response-repetition cost in task-switch trials). Correspondingly, the between-experiment ANOVA, while not showing a general difference in error rates across Experiments 1A and 1B (5.0% vs. 4.9%; *F*(1, 98) = 0.054, *p* = .817, η_p_^2^ = .001), revealed a significant three-way interaction (*F*(1, 98) = 23.153, *p <* .001, η_p_^2^ = .191). Hence, opposing the pattern of the three-way interaction in the RTs, the interaction between task transition and response repetition in the error rates was significantly smaller in the three-choice (vs. two-choice) task. The two-way interactions between (a) experiment and task transition and (b) experiment and response transition were not significant (*F*s < 1.2, *p*s > .28).

In summary, the three-way interaction in RT tended to be larger in the three-choice (vs. two-choice) task while the opposite pattern appeared in error rates. This outcome does not reveal a consistent effect of the number of response alternatives (two vs. three) on the interaction pattern. Critically, the main finding of Experiment 1B is the significant response-repetition costs in task-switch trials, which appeared in both RTs and error rates. As we explained earlier, the bias to switch responses account does not predict this effect while the other accounts do.

## General discussion

We examined the influence of the number of response alternatives on response-repetition effects in task switching. In line with the literature (for reviews, see, e.g., Gade et al., 2014; Koch et al., 2018b), we replicated the basic interaction between task repetition and response repetition in the two-choice task of Experiment 1A. Specifically, we observed a response-repetition benefit in task-repeat trials but a response-repetition cost in task-switch trials. Consistent with prior findings from tasks involving univalent stimuli, the response-repetition cost was more robust in error rate than in RT (Kleinsorge, 1999; Rogers & Monsell, 1995, Experiment 4). Critically, we also observed a response-repetition cost in the three-choice task of Experiment 1B. This outcome appears to favor the response inhibition and associative interference accounts over the bias to switch responses account.

### Influence of the number of response alternatives

The primary effect of increasing the number of response alternatives was an increase of RT with more alternatives. This increase of RT is consistent with the venerable Hick-Hyman law (Hick, 1952; Hyman, 1953) relating RT to the amount of information to be processed (see Proctor & Schneider, 2018, for a historical review). This effect is likely due to two, probably related, processes. First, with more response alternatives there is more associative interference during response retrieval, slowing down response selection (Schneider & Anderson, 2011). Second, more responses may increase the probability that an incorrect response alternative reaches the selection threshold, so that the response decision criterion needs to be adjusted to reach acceptable accuracy (Usher et al., 2002). In our study, we found almost identical overall accuracy in the two- and three-choice tasks (error rates of 5.0% vs. 4.9%, respectively). This suggests that the response criterion was more cautious in the three-choice task than in the two-choice task, resulting in prolonged response selection times and, therefore, increased RT in the three-choice task relative to the two-choice task.

Given the effectiveness of the number-of-response-alternatives manipulation on overall RT, it is notable that this manipulation exerted opposite effects on the pattern of response-repetition effects in RT and error rates. Specifically, while we observed a slightly more pronounced interaction of response transition and task transition in the RT data of the three-choice task relative to the two-choice task, we observed the opposite in the error rate data. As described above, an adjustment of the response criterion probably led participants to respond more cautiously in the three-choice task. This would drive some of the variance out of the error rates and into RT, thereby accentuating RT effects that would otherwise have been spread more equally across RT and error rate. For the error rate data, this would reduce both (a) the response-repetition cost in task switches and (b) the response-repetition benefit in task repetitions, just as we observed. The net effect would be a flattened interaction pattern in the error rates and an accentuated pattern of interaction in the RTs, which is what we found. Previous studies have already used more than two response alternatives in task switching (e.g., Kikumoto & Mayr, 2020), but they did not assess the influence of this variable directly. Based on the present findings, we conclude that, overall, varying the number of response alternatives has no consistent effect on the interaction pattern of task switching and response repetition when the RT and error rate data are considered jointly.

### Response-repetition costs in task-switch trials

As described in the *Introduction*, the three main accounts of the task transition × response transition interaction make distinct predictions regarding response-repetition costs in task switches. The bias to switch responses account assumes that a task switch biases the system toward a response switch. In a two-choice task, this facilitates the single response alternative and thereby leads to a response-repetition cost. In a three-or-more-choice task, however, a task switch cannot facilitate a *specific* alternative response. Consequently, there is no reason to predict response-repetition costs as with two alternatives. Our data rule out a strong interpretation of this account. We observed significant response-repetition costs in the task-switch trials of the three-choice task in Experiment 1B in both RT and error rates.

One might, however, entertain an alternative view of the bias to switch responses account. Here, participants randomly prepare one of the two alternative response options when the task switches. This strategy predicts a response-switch benefit in about half the trials due to preparing the correct response and a response-switch cost in the other half of trials due to preparing the incorrect response. Thus, on average, this strategy should neither facilitate nor hinder performance when the response switches in task-switch trials. However, as response repetitions in task-switch trials are always relatively unprepared, the net effect could still be a small response-repetition cost, which is about half the size as when there are two response alternatives (i.e., as in Experiment 1A). Critically, using such a strategy in task-switch trials should produce a bimodal RT distribution when the response switches, which consists of relatively fast, “correctly prepared” response switches and relatively slow, “incorrectly prepared” response switches. Consequently, one would expect greater RT variability in task-switch trials when the response (a) switches versus (b) repeats. Exploratory analyses, however, revealed that this was not the case (*M* = 710 ms, *SD* = 99.97 ms for response switches and *M* = 723 ms, *SD* = 100.92 ms for response repetitions, respectively). These findings weigh against the alternative view of the bias to switch responses account.

Finally, one might entertain a second alternative view of the bias to switch responses account wherein a task switch biases participants to prepare both alternative responses (Kleinsorge et al., 2001). As we explained in the *Introduction*, an influential variant of this view posits that tasks are organized hierarchically, such that changing a task-relevant feature at a higher level of the hierarchy (e.g., the task set, the color of the stimulus, etc.) facilitates a response alternation at lower levels (Kleinsorge, 1999). Such a parallel response preparation is possible with the current setup because the response keys are operated with different fingers. To prevent such a preparation, and thereby test this account of our findings, one could employ a task wherein responding to each of several stimuli always requires moving the same finger (e.g., the right index finger) from a home key to a stimulus-specific key. In such a task, it is not possible to prepare more than one finger response at a time, because participants can use only one finger to respond. Yet, while we cannot exclude this interpretation of the response-repetition costs in our three-choice task, prior results weigh against it. Specifically, while alternating between different task-relevant stimulus features (e.g., different colors) is associated with robust response-repetition costs in two-choice tasks, such costs are reduced or eliminated in three-or-more-choice tasks (e.g., Fletcher & Rabbitt, 1978; Weissman et al., 2023). Such costs should not be reduced, however, if changing a task-relevant feature at a higher level of the hierarchy biases participants to prepare *both* alternative responses. Thus, these findings weigh against this alternative view.

In the context of their “generalized switching account,” Kleinsorge and Heuer (1999) also discussed strategic response biases. These authors examined the generalization of this account to “three-valued task dimensions” (Kleinsorge et al., 2001). They manipulated the number of judgment tasks, response mappings, and responses as well as the respective transition levels of these three variables. In their Experiment 1, with two alternatives, they found that switches at higher levels (e.g., task switches) biased the system toward switches at lower levels (e.g., response switches). However, in their Experiment 2 with three alternatives, switches at higher levels influenced switches at the lower level of the response mapping in a more additive (less interactive) manner, as predicted by their generalized switching account. Yet, when focusing on the repeated response mapping conditions, which are more similar to our experimental conditions, they still found a complete crossover interaction for task switching and response repetition in the error rates (just as we did) and a similar but less pronounced interaction pattern for the RTs (i.e., the response-repetition costs in task switches seemed to be very small at best). Given the complexity of Kleinsorge et al.’s (2001) experimental design and the relatively small sample size they employed (n = 12), their findings may not be conclusive. However, the present study, which employs a simpler experimental design and provides relatively high statistical power, appears to confirm their findings. More specifically, our findings support the view that varying the number of response alternatives has no consistent influence on response-repetition effects in task switching. This outcome is not easy to reconcile with the bias to switch responses account.

### Potential relevance to other research on sequential action control

Studies of task switching investigate the abstract representations on which action control is based (such as stimulus category instead of the specific stimulus identity). Given this aim, it is important to note that response-switch biases signaled by stimulus change may play a role in simpler action control paradigms (Fletcher & Rabbitt, 1978) including those used to examine the binding and retrieval approach (Frings et al., 2020). For example, the number of response alternatives influences the pattern of repetition effects in choice RT tasks (e.g., Akçay & Hazeltine, 2007; see Hazeltine et al., 2022). Moreover, in recent work using the “partial repetition cost” paradigm (Hommel, 1998; see also Huffman et al., 2020), we observed an attenuated pattern of partial repetition costs when increasing the number of alternatives from two to four (see Weissman et al., 2023). Thus, although changing an abstract task set may not be sufficient for a response-switch bias heuristic to operate, such a heuristic might still be relevant when a concrete stimulus feature changes or repeats. Future work could detail the specific predictions of the extant theoretical accounts and examine whether these differ with respect to the level of abstractness to which the corresponding control processes refer. Moreover, while the present data seem to be generally consistent with feature binding and response inhibition accounts, these accounts do not seem to make strong assumptions about how the response criterion is adjusted as a function of the number of alternatives, leaving room for future theoretical development.

## Conclusion

The present findings show that varying the number of response alternatives exerts a moderate influence at best on response-repetition effects in task switching. This outcome appears more consistent with the response inhibition and feature binding accounts of such response-repetition effects than with the bias to switch responses account.

## References

[CR1] Akçay Ç, Hazeltine E (2007). Conflict monitoring and feature overlap: Two sources of sequential modulations. Psychonomic Bulletin & Review.

[CR2] Altmann EM (2011). Testing probability matching and episodic retrieval accounts of response repetition effects in task switching. Journal of Experimental Psychology: Learning, Memory, and Cognition.

[CR3] Anwyl-Irvine AL, Massonnié J, Flitton A, Kirkham N, Evershed JK (2020). Gorilla in our midst: An online behavioral experiment builder. Behavior Research Methods.

[CR4] Druey MD (2014). Stimulus-category and response-repetitions effects in task switching: An evaluation of four explanations. Journal of Experimental Psychology: Learning, Memory, and Cognition.

[CR5] Druey MD, Hübner R (2008). Response inhibition under task switching: Its strength depends on the amount of task-irrelevant response activation. Psychological Research.

[CR6] Egner T (2007). Congruency sequence effects and cognitive control. Cognitive, Affective, & Behavioral Neuroscience.

[CR7] Faul, F., Erdfelder, E., Lang, A.G., & Buchner, A. (2007). G*Power 3: A flexible statistical power analysis program for the social, behavioral, and biomedical sciences. *Behavior Research Methods, 39*, 175–191. 10.3758/bf03193146 17695343

[CR8] Fletcher BC, Rabbitt PM (1978). The changing pattern of perceptual analytic strategies and response selection with practice in a two-choice reaction time task. The Quarterly Journal of Experimental Psychology.

[CR9] Frings C, Hommel B, Koch I, Rothermund K, Dignath D, Giesen C, Kiesel A, Kunde W, Mayr S, Moeller B, Möller M, Pfister R, Philipp AM (2020). Binding and Retrieval in Action Control (BRAC). Trends in Cognitive Sciences.

[CR10] Gade M, Schuch S, Druey MD, Koch I, Grange J, Houghton GH (2014). Inhibitory control in task switching. Task switching and cognitive control.

[CR11] Grzyb KR, Hübner R (2013). Excessive response-repetition costs under task switching: How response inhibition amplifies response conflict. Journal of Experimental Psychology: Learning, Memory, and Cognition.

[CR12] Hazeltine, E., Koch, I., & Weissman, D. (2022). *Comparing partial repetition costs in two- and four-choice tasks: Evidence for the use of signaling by abstract codes*. Manuscript submitted for publication. 10.1037/xlm0001318 38095953

[CR13] Hick WE (1952). On the rate of gain of information. Quarterly Journal of Experimental Psychology.

[CR14] Hommel, B. (1998). Event files: Evidence for automatic integration of stimulus-response episodes. *Visual Cognition, 5*, 183–216.

[CR15] Hommel B, Müsseler J, Aschersleben G, Prinz W (2001). The theory of event coding (TEC): A framework for perception and action planning. Behavioral and Brain Sciences.

[CR16] Hübner R, Druey MD (2006). Response execution, selection, or activation: What is sufficient for response-related repetition effects under task shifting?. Psychological Research.

[CR17] Huffman, G., Hilchey, M. D., Weidler, B. J., Mills, M., & Pratt, J. (2020). Does feature-based attention play a role in the episodic retrieval of event files? *Journal of Experimental Psychology: Human Perception and Performance, 46*, 241–251. 10.1037/xhp0000709 32077740

[CR18] Hyman R (1953). Stimulus information as a determinant of reaction time. Journal of Experimental Psychology.

[CR19] Kandalowski S, Seibold J, Schuch S, Koch I (2020). Examining binding effects on task switch costs and response-repetition effects: Variations of the cue modality and stimulus modality in task switching. Attention, Perception & Psychophysics.

[CR20] Kikumoto A, Mayr U (2020). Conjunctive representations that integrate stimuli, responses, and rules are critical for action selection. Proceedings of the National Academy of Sciences.

[CR21] Kiesel A, Steinhauser M, Wendt M, Falkenstein M, Jost K, Philipp AM, Koch I (2010). Control and interference in task switching – A review. Psychological Bulletin.

[CR22] Kleinsorge T (1999). Response repetition benefits and costs. Acta Psychologica.

[CR23] Kleinsorge T, Heuer H (1999). Hierarchical switching in a multi-dimensional task space. Psychological Research.

[CR24] Kleinsorge T, Heuer H, Schmidtke V (2001). Task-set reconfiguration with binary and three-valued task dimensions. Psychological Research.

[CR25] Koch I, Kiesel A, Kiesel A, Johannsen L, Koch I, Müller H (2022). Task switching: Cognitive control in sequential multitasking. Handbook of human multitasking.

[CR26] Koch I, Schuch S, Vu K-PL, Proctor RW (2011). Response-repetition effects in task switching – dissociating effects of anatomical and spatial response discriminability. Acta Psychologica.

[CR27] Koch I, Frings C, Schuch S (2018). Explaining response-repetition effects in task switching: evidence from switching cue modality suggests episodic binding and response inhibition. Psychological Research.

[CR28] Koch I, Poljac E, Müller H, Kiesel A (2018). Cognitive structure, flexibility, and plasticity in human multitasking – An integrative review of dual-task and task-switching research. Psychological Bulletin.

[CR29] Meiran N (2000). Modeling cognitive control in task-switching. Psychological Research.

[CR30] Monsell S (2003). Task switching. Trends in Cognitive Sciences.

[CR31] Quinlan PT (1999). Sequential effects in auditory choice reaction time tasks. Psychonomic Bulletin & Review.

[CR32] Proctor RW, Schneider DW (2018). Hick's law for choice reaction time: A review. Quarterly Journal of Experimental Psychology.

[CR33] Rogers RD, Monsell S (1995). Costs of a predictable switch between simple cognitive tasks. Journal of Experimental Psychology: General.

[CR34] Schacherer, J., & Hazeltine, E. (2022). Response-repetition costs reflect changes to the representation of an action. *Psychonomic Bulletin & Review*, *29*, 2146–2154. 10.3758/s13423-022-02115-y 35618943

[CR35] Schneider DW, Logan GD (2005). Modeling task switching without switching tasks: A short-term priming account of explicitly cued performance. Journal of Experimental Psychology: General.

[CR36] Schneider DW, Anderson JR (2011). A memory-based model of Hick's law. Cognitive Psychology.

[CR37] Schuch S, Koch I (2004). The costs of changing the representation of action: response repetition and response-response compatibility in dual tasks. Journal of Experimental Psychology: Human Perception and Performance.

[CR38] Schuch S, Koch I (2006). Task switching and action sequencing. Psychological Research.

[CR39] Schuch S, Koch I (2010). Response-repetition effects in task switching with and without response execution. Acta Psychologica.

[CR40] Seibold JC, Koch I, Nolden S, Proctor RW, Vu K-PL, Schuch S (2019). Response repetitions in auditory task switching: The influence of spatial response distance and of the response-stimulus interval. Acta Psychologica.

[CR41] Usher M, Olami Z, McClelland JL (2002). Hick's law in a stochastic race model with speed-accuracy tradeoff. Journal of Mathematical Psychology.

[CR42] Weissman D, Hawks Z, Egner T (2016). Different levels of learning interact to shape the congruency sequence effect. Journal of Experimental Psychology: Learning, Memory, and Cognition.

[CR43] Weissman, D., Grant, L., Koch, I., & Hazeltine, E. (2023). Sequential effects in action control: Partial repetition costs index a mixture of binding and signaling. *Attention, Perception & Psychophysics, 85, *505–524. 10.3758/s13414-022-02539-7 PMC9937548 35864294

[CR44] Williams JA (1966). Sequential effects in disjunctive reaction time: Implications for decision models. Journal of Experimental Psychology.

